# Development of Open-Field Behaviour in the Medaka, *Oryzias latipes*

**DOI:** 10.3390/biology9110389

**Published:** 2020-11-10

**Authors:** Tyrone Lucon-Xiccato, Francesca Conti, Felix Loosli, Nicholas S. Foulkes, Cristiano Bertolucci

**Affiliations:** 1 Department of Life Sciences and Biotechnology, University of Ferrara, 44121 Ferrara, Italy; lcntrn@unife.it (T.L.-X.); francesca03.conti@edu.unife.it (F.C.); 2 Institute of Biological and Chemical Systems, Biological Information Processing (IBCS-BIP), Karlsruhe Institute of Technology, Hermann-von-Helmholtz Platz 1, 76344 Eggenstein-Leopoldshafen, Germany; felix.loosli@kit.edu (F.L.); nicholas.foulkes@kit.edu (N.S.F.)

**Keywords:** anxiety, behavioural models, exploration, fish behaviour, Japanese rice fish, novel tank test, medaka

## Abstract

**Simple Summary:**

Animal models play an important role in research on behaviour and its impairment. Fish larvae allow researchers to conduct experiments on large samples in just a few days and with small-scale experimental infrastructure, substantially increasing research output. However, several aspects of larval biology, including their behaviour, are frequently unknown. Our study has demonstrated that the most important behavioural paradigm for studying anxiety and stress in animals, the open-field test, can be used in the larvae of an important fish genetic model, the medaka. This finding will allow researchers to develop models to study anxiety and stress disorders based on medaka larvae.

**Abstract:**

The use of juvenile and larval fish models has been growing in importance for several fields. Accordingly, the evaluation of behavioural tests that can be applied to larvae and juveniles is becoming increasingly important. We tested medaka at four different ages (1, 10, 30, and 120 dph) in the open field test, one of the most commonly used behavioural assays, to investigate its suitability for larvae and juveniles of this species. We also explored ontogenetic variation in behaviour during this test. On average, adult 120-day-old medaka showed higher locomotor activity in terms of distance moved compared with younger fish. Our analysis suggests that this effect was derived from both quantitative changes in locomotion related to the ontogenetic increase in fish size as well as qualitative changes in two aspects of locomotor behaviour. Specifically, time spent moving was similar between 1- and 10-day-old medaka, but progressively increased with development. In addition, we revealed that adult medaka showed constant levels of activity, whereas younger medaka progressively reduced their activity over the course of the entire experiment. The thigmotaxis behaviour typically used to assess anxiety in the open field test emerged at 120 days post-hatching, even though a difference in the temporal pattern of spatial preference emerged earlier, between 10 and 30 days post-hatching. In conclusion, some measures of the open field test such as total distance moved allow behavioural phenotyping in the medaka of all ages, although with some degree of quantitative and qualitative developmental variation. In contrast, immature medaka appear not to exhibit thigmotactic behaviour.

## 1. Introduction

Recent years have witnessed a substantial increase in the use of teleosts as models in many research fields, including behavioural sciences [1,2]. As seen with other vertebrate models, the use of fish would benefit from rapid, reliable, and high-throughput tests for behavioural phenotyping. Accordingly, great effort has been devoted to adapting behavioural tests from other models or to specifically develop behavioural tests for fish [2]. The open-field test (OFt) is one of the most frequently used behavioural tests in animal research. It has been developed to measure anxiety, activity, exploration, and related phenotypes in rodents [3]. The subject is inserted in a novel, empty arena. Then, behavioural variables such as the amount of activity/locomotion and spatial preferences are recorded, often with the use of automatic tracking [4]. Several versions of the OFt are now available for fish [5,6], consisting of measuring the behavioural response to a novel aquarium. These aquatic OFt are routinely adopted to screen mutant genotypes and the effects of drugs [7,8].

The great impetus in the research on fish models is at least in part motivated by their rapid-life cycle and low maintenance costs. In sharp contrast with mammalian models, most fish used in research have juveniles or even larvae stages that are fully independent from their parents, swim freely, and exhibit a rather complex behavioural repertoire [9,10,11,12]. As a consequence, it is possible to conduct experiments in subjects of just a few days old and small size, significantly increasing the rate of experimental analysis and reducing the scale of required experimental infrastructure [13]. This progress has been associated with recent advances in technologies that have enabled the dissection of the genetic and physiological basis of various aspects of behaviour. These advances have permitted, for example, studying dynamic changes in gene expression in vivo in juvenile and larval fish models and recording whole brain neural activation of freely swimming subjects [14,15]. However, the use of larvae and/or juveniles is currently constrained by a lack of knowledge of their behaviour, with particular reference to ontogenetic changes and differences from adults. For example, in the zebrafish, *Danio rerio*, and the Eastern mosquitofish, *Gambusia holbrooki*, the OFt behaviour changes dramatically during ontogeny [16,17], demonstrating that it is not possible to simply adopt the same tests initially developed for adult fish.

The medaka, *Oryzias latipes*, is a teleost species increasingly used in various research fields, including genetic [18,19], biomedical sciences [20], and neurosciences [21,22,23]. A few studies have exploited the use of larval or juvenile medaka [24], however, to date, the behavioural characterization of medaka during ontogeny has not been conducted. In particular, the OFt has been exploited for pharmacological [25], toxicological [26], and cognitive studies in medaka, but only in adults [27]. In this study, we investigated medaka behaviour in the OFt through ontogeny. In particular, we asked (i) whether the OFt can be used in larvae and juvenile medaka and (ii) whether behavioural differences are observed between medaka of different ages.

## 2. Materials and Methods

### 2.1. Subjects

We tested 64 medaka divided into the following age groups: 16 larvae at 1 dph (days post-hatching), 16 larvae at 10 dph, 16 juveniles at 30 dph, and 16 adults at 120 dph. Subjects were matched for size within each age group. We obtained the subjects from wild-type medaka (the isogenic inbred ‘iCab’ strain) maintained in the fish facility of University of Ferrara. These fish were kept in standard 200 L glass aquaria equipped with biological and mechanical filters and maintained at 28 ± 1 °C under a 14 h light/10 h dark (LD 14:10) photoperiod. Each aquarium hosted approximately 30 fish of both sexes, which could spontaneously breed. On a daily basis, eggs were removed from the females with the help of tweezers. Then, the eggs were washed and placed in Petri dishes filled with ERM 1× (embryo raising medium; 10×: 10 g NaCl, 0.3 g KCl; 0.4 g CaCl_2_·2 H_2_O; 1.63 g MgSO_4_·7 H_2_O; 170 mL 1 M Hepes pH 7.3 for 1 L) and three drops of methylene blue dye. Each Petri dish contained 50 eggs and was kept in an incubator set at 28 °C and under LD 14:10. Each day, 50% of the medium was substituted with fresh medium until hatching.

The medaka typically hatched within 8–10 days. Each morning, hatched larvae were collected and moved into rectangular glass aquaria (capacity: 1.8 L), filled with 50 mL of FW 1× (fish water; 50×: 25 g Instant Ocean, 39.25 g CaSO_4_ and 5 g NaHCO_3_ for 1 L) and kept in the incubator as described before. The larvae were fed twice a day with dry food (Micron Nature, Sera GmbH, Immenhausen, Germany). Fifteen days after hatching, the larvae were transferred into larger glass tanks (2 L). Thirty-one days after hatching, the fish were moved into standard maintenance tanks and their diet was complemented with live *Artemia salina* nauplii. We randomly assigned larvae hatched to the testing groups of different ages.

### 2.2. Open-Field Test

Our OFt was performed following the most common procedure used in fish. We tested each subject individually in a white plastic square arena. Transportation of the subjects to the arena was performed using a container, which reduced stress for the subjects, compared with transportation with a net. The arena was filled with ERM, FW, or standard aquarium water according to the subject’s developmental stage. Because medaka size varies with age, the size of the arena was scaled, according to the age of the subjects. Based on previous studies in adult and larvae fish [16,28], the size of the arena was varied as follows:

1 dph: 8 cm × 8 cm, filled with 2.5 cm of ERM;
10 dph: 8 cm × 8 cm, filled with 2.5 cm of ERM;
30 dph: 12 cm × 12 cm, filled with 4 cm of FW;
120 dph: 40 cm × 40 cm, filled with 12 cm of water.

**Figure 1:**
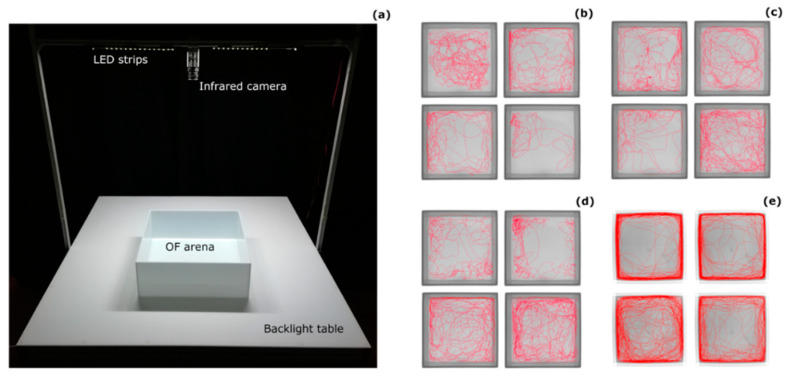
Open-field paradigm for medaka. (**a**) Experimental set up and tracks of 16 randomly-chosen subjects that illustrate differences in the open field behaviour between (**b**) 1 dph, (**c**) 10 dph, (**d**) 30 dph, and (**e**) 120 dph medaka.

The arena was placed on a backlight table illuminated with infrared LEDs (λ > 980 nm; Noldus Information Technology, Wageningen, The Netherlands). The experimental room was kept in darkness, with warm-white LED strips placed 1 m above the table illuminating the arena. An infrared camera (Monochrome GigE camera, Basler, Germany; resolution: 1280 × 1024) was placed 1 m above the arena to video record the experiments at 5 frames per second. A computer running the EthoVision XT software (Noldus Information Technology, Wageningen, The Netherlands) and connected to the camera tracked the movements of the fish in the arena (Figure 1). The behaviour of each fish was collected for 30 min after the release into the arena. The software calculated three dependent variables typically used to describe fish behaviour with the OFt: distance moved, time spent moving, and time spent in the centre of the arena [29,30]. For time spent swimming, the subjects’ body length/second was used as a threshold for movement, allowing us to analyse the subjects’ behaviour controlling for their size. Time spent in the centre of the arena was assessed with a threshold of one body length from the edges [28]. All these variables were measured for each minute of the test to allow analysis of the temporal pattern of behaviour.

### 2.3. Statistical Analysis

Analysis was performed with R Statistical software version 4.0.1 (The R Foundation for Statistical Computing, Vienna, Austria, http://www.r-project.org). Distance moved was log-transformed before the analysis. For each behavioural variable, first, a one-way analysis of variance (ANOVA) was performed on the overall performance of the subjects. Age was defined as a fixed effect and Tukey post-hoc tests were used to investigate significant effects. Then, a second analyses was run on the data split into 1 min time bins. Each age was analysed separately. Linear mixed-effects models were used, fitted with the *lme* function of the *nlme* R package. This analysis allowed us to detect temporal changes in fish behaviour. The dataset of the experiment is available as supplementary material (Data S1).

### 2.4. Ethical Approval

Experiments were conducted in accordance with Italian law (Italy, D.L. 4 Marzo 2014, n. 26). The Ethical Committee of University of Ferrara reviewed and approved all the experimental procedures (protocol n. CB/01-2019, 8 November 2019). At the end of the experiments, all subjects were released into stock tanks.

## 3. Results

### 3.1. Distance Moved

**Figure 2:**
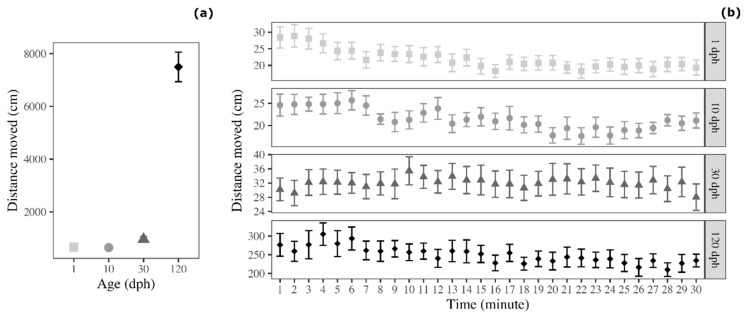
Distance moved by medaka in the open-field test (**a**) as a function of age and (**b**) as a function of age and time (30 1 min time bins). Points represent means of the different age groups (squares = 1 dph, circles = 10 dph, triangles = 30 dph, and rhombuses = 120 dph) and error bars represent standard errors (*n* = 16 per age group).

Analysis on the average distance moved revealed a significant effect of age (F_3__,60_ = 137.650, *p* < 0.001; Figure 2a). This effect was due to the fact that 120 dph medaka moved for greater distances compared with 1 dph medaka (Tukey post-hoc test: t = 17.263, *p* < 0.001), 10 dph medaka (t = 17.216, *p* < 0.001), and 30 dph-medaka (t = 14.827, *p* < 0.001; Figure 1). Conversely, the three groups of immature medaka (1, 10, and 30 dph) did not show significant differences in terms of distance moved during the OFt (Tukey post-hoc test: 1 dph versus 10 dph: t = 0.047, *p* > 0.999; 1 dph versus 30 dph: t = 2.463, *p* = 0.081; 10 dph versus 30 dph: t = 2.388, *p* = 0.090).

The analysis of the temporal trend, which is often used to evaluate habituation to the novel tank, revealed that distance moved varied across testing time. In particular, it decreased for 1 dph medaka (F_1__,463_ = 87.157, *p* < 0.001), 10 dph medaka (F_1__,463_ = 36.666, *p* < 0.001), and 120 dph medaka (F_1__,463_ = 17.234, *p* < 0.001), and the same trend was observed for 30 dph medaka (F_1__,463_ = 3.635, *p* = 0.057; Figure 2b).

### 3.2. Time Spent Moving

**Figure 3:**
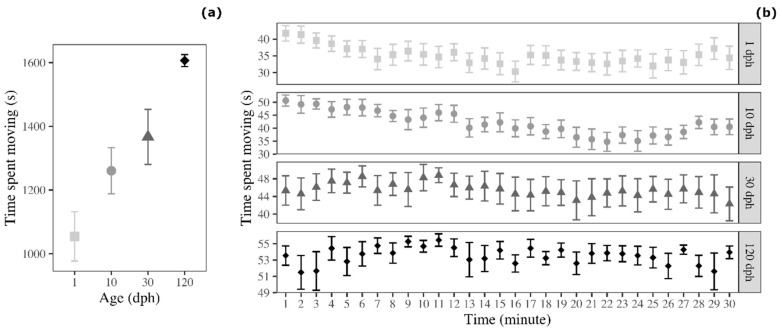
Time spent moving by medaka in the open-field test (**a**) as a function of age and (**b**) as a function of age and time (30 1 min time bins). Points represent means of the different age groups (squares = 1 dph, circles = 10 dph, triangles = 30 dph, and rhombuses = 120 dph) and error bars represent standard errors (*n* = 16 per age-group).

Because the distance moved was also affected by the size of the fish, we analysed a second activity variable controlled for such factor, the time spent moving with the threshold for movement set to fish body length. The analyses revealed that the time spent moving varied significantly with fish age (ANOVA: F_3__,60_ = 11.081, *p* < 0.001; Figure 3a). In detail, 120 dph medaka spent more time moving compared with 1 dph medaka (Tukey post-hoc test: t = 5.657, *p* < 0.001) and 10 dph medaka (t = 3.545, *p* = 0.004). In addition, 30 dph medaka spent more time moving compared with 1 dph medaka (t = 3.200, *p* = 0.011). The post-hoc comparisons between the remaining age groups were not significant (1 dph versus 10 dph: t = 2.112, *p* = 0.160; 10 dph versus 30 dph: t = 1.088, *p* = 0.698; 30 dph versus 120 dph: t = 2.457, *p* = 0.078).

We detected an age-difference in the temporal trend indicated of time spent moving. Here, 1 dph medaka (F_1__,463_ = 33.661, *p* < 0.001), 10 dph medaka (F_1__,463_ = 97.285, *p* < 0.001), and 30 dph medaka (F_1__,463_ = 8239, *p* = 0.004) decreased the time spent moving over testing time, but time spent moving was constant for 120 dph medaka (F_1__,463_ = 0.359, *p* = 0.549; Figure 3b).

### 3.3. Time Spent in the Centre of the Arena

**Figure 4:**
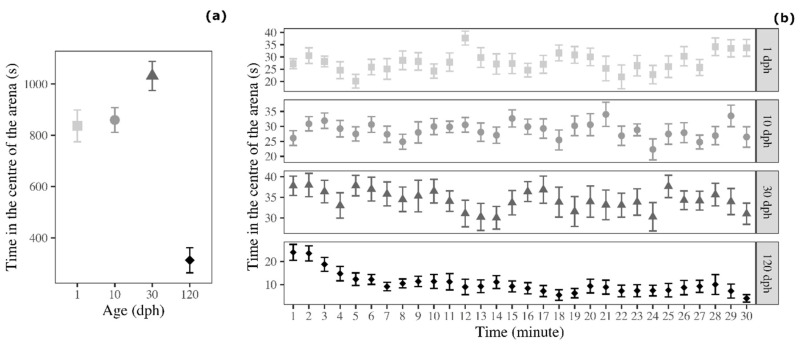
Time spent by medaka in the centre of the arena in the open-field test (**a**) as a function of age and (**b**) as a function of age and time (30 1 min time bins). Points represent means of the different age groups (squares = 1 dph, circles = 10 dph, triangles = 30 dph, and rhombuses = 120 dph) and error bars represent standard errors (*n* = 16 per age-group).

There was a significant effect of age on the time spent in the centre of the arena (F_3,60_ = 32.736, *p* < 0.001; Figure 4a). Tukey post-hoc test indicated that 120 dph medaka showed avoidance of the centre of the arena compared with 1 dph medaka (t = 6.822, *p* < 0.001), 10 dph medaka (t = 7.122, *p* = 0.004), and 30 dph medaka (t = 9.360, *p* < 0.001; Figure 1). The three younger groups of medaka (1, 10, and 30 dph) did not showed differences in this variable (Tukey post-hoc test: 1 dph versus 10 dph: t = 0.300, *p* = 0.991; 1 dph versus 30 dph: t = 2.537, *p* = 0.064; 10 dph versus 30 dph: t = 2.238, *p* = 0.125).

The analysis of the temporal trend indicated that time spent in the centre of the arena significantly decreased over testing time for 30 dph medaka (F_1,463_ = 4.635, *p* < 0.001) and 120 dph medaka (F_1,463_ = 61.857, *p* < 0.001). Conversely, 1 dph medaka (F_1,463_ = 2.891, *p* = 0.090) and 10 dph medaka (F_1,463_ = 0.671, *p* = 0.413; Figure 4b) did not vary time spent in the centre of the arena during the OFt.

## 4. Discussion

In this study, we tested mekada in the open-field test at four different ages: 1, 10, 30, and 120 dph. The distance swum by medaka in the open-field arena was similar during the first month of life, but it increased approximately ten times when fish reached adulthood. However, there were no substantial differences between different-aged medaka in the temporal pattern of this variable, which tended to decrease across testing time irrespectively of the age of the fish. Usually, the temporal pattern of activity during the experiment is considered an important indicator of habituation to the novel environment [27,31]. Therefore, habituation occurred similarly in different-aged medaka. The similar habituation pattern in the four age groups additionally suggests that the greater distance moved by adult fish was a quantitative rather than qualitative effect. It could be ascribed to differences in swimming performance between the age groups. A key determinant of fish swimming performance is body size [32], which covaries with the age of the fish. We first attempted to reduce the impact of this variable by increasing the size of the OF arena with the age of the subjects. In addition, we assessed the potential effect of body size by comparing the distance moved by medaka with a measure of time spent moving corrected for subjects’ body length. Considering the latter variable, medaka still showed an ontogenetic increase in the activity in the OF. However, the behaviour of adult fish was less divergent compared with that of younger fish and there seemed to be a more constant ontogenetic change (Figure 1). It can be concluded that adult fish swim more in the OFt, in part because adults are larger and in part because of an intrinsic variation in the tendency to swim, i.e., adults spend most of their time swimming.

An effect of age on swimming has been detected when comparing adult and larval zebrafish [33]. Such an effect has been attributed to differences in general routine activity because the fish were acclimatised to the observation tank before the activity recording [33]. However, in our study, we tested medaka in an unfamiliar environment, suggesting that findings in medaka and in zebrafish likely reflect a different mechanism. The increased time adult medaka spent swimming in the OF might be related to anxiety and stress levels, including those generated by manipulation before the test [34]. Two diverse hypotheses have been proposed to link anxiety and stress to swimming in the OFt according to the species studied. More anxious individuals swim more intensively in the attempt to escape from the OF arena [28,30] or, alternatively, they perform more freezing behaviour, resulting in reduced swimming [35]. As anxiety and stress are expected to be higher for smaller compared with larger fish [36], the second hypothesis seems more suitable to account for our results. Finally, age-differences in exploratory tendency may also be relevant. For example, in zebrafish and guppies, adult fish are more explorative compared with larvae and juveniles [37,38]. A similar ontogenetic variation may explain our results in terms of adult medaka being more explorative in the novel environment, and thus swimming more compared with younger fish.

The analysis of subjects’ spatial position revealed a different pattern compared with the activity measures of above. Adult fish spent much more time at the edges of the arena compared with younger medaka, a behaviour usually referred to as thigmotaxis. At one month of age, medaka showed only some features of adults’ spatial behaviour, such as a decrease in the time spent in the centre of the arena over the testing period. Therefore, only as adults do medaka fully show the typical thigmotactic behaviour observed in other fish species [39]. In the zebrafish, larvae tend to avoid the centre of the open field arena since the first days of life, and this behaviour is commonly used to assess individuals’ anxiety levels [40,41]. Several explanations may account for the large behavioural differences between adult and immature medaka. A role of swimming performance seems unlikely because the OF arena was matched with the size of the fish. Moreover, explanations based on swimming performance assume that larger fish could not explore the entire arena because of limited swimming capacities; however, larger fish are known to have greater swimming performance [32]. Another possibility is that young medaka do not respond to the novel environment or do not show anxiety. However, this explanation is not consistent with the behavioural trends observed for distance moved, i.e., a decrease over time, indicating habituation was observed for all medaka age groups. Therefore, it seems possible that immature medaka recognised the novelty in the testing arena, but did not respond to it with altered spatial behaviour. In a cricket species, a link has been demonstrated between thigmotaxis and spatial cognition capacities [42]. It is possible that the medaka cognitive system is still immature at the larval and juvenile stages, and only after its maturation in adult fish would it fully perceive the environment, and thereby direct thigmotactic behaviour. A detailed investigation of how the development of the nervous system underlies the maturation of cognitive abilities in medaka clearly promises to be an important topic of future investigation. Additionally, it will be important to assess the validity of anxiety measures that do not involve spatial behaviour in medaka larvae, such as the response to tactile stimulation [43], the response to predator odours and conspecific alarm cues [44], and the response to novel chemicals [37], which are currently exploited in other fish larvae models.

## 5. Conclusions

As a whole, our study supports the utility of the OFt to screen behaviour in larvae and juvenile medaka, but only when particular activity parameters are considered (e.g., distance moved, time spent moving). Caution should be exerted in interpreting differences between experiments with different-aged subjects because of the potential influence of body size. In sharp contrast, evidence gathered in this study poses concerns over the use of thigmotaxis and other spatial position measures to study anxiety in larval and juvenile medaka. Other types of anxiety measures that do not rely on spatial behaviour may be more appropriate for implementing anxiety tests in larval medaka.

## Supplementary Materials

The following are available online at https://www.mdpi.com/2079-7737/9/11/389/s1, Data S1: dataset of the open-field experiment in groups of medaka with different age.
