# Supplementary material for: Development of Open-Field Behaviour in the Medaka, Oryzias latipes
Source: Biology (Basel). 2020 Nov 10;9(11):389. doi: 10.3390/biology9110389 (PMC7696969; doi:10.3390/biology9110389)
Supplement: Supplementary file 1 [file biology-09-00389-s001.pdf]

subject	age	distance.moved	time.moving	time.spent.centre
medaka 1	dph001	345,21409	822,16	1094,72
medaka 2	dph001	583,60454	1073,16	755,8
medaka 3	dph001	701,817	1232,6	825,64
medaka 4	dph001	221,66363	328,72	1421,92
medaka 5	dph001	642,1518	1062,76	775,28
medaka 6	dph001	857,5027	1318,48	911,76
medaka 7	dph001	466,52786	907,48	878,2
medaka 8	dph001	632,8894	1176,16	950,8
medaka 9	dph001	562,777	824,68	1064,4
medaka 10	dph001	661,2473	1203,76	505,72
medaka 11	dph001	856,7467	1359,52	394,32
medaka 12	dph001	1080,6879	1453,32	780,48
medaka 13	dph001	1050,5848	1526,36	951,56
medaka 14	dph001	353,45822	723,32	739,08
medaka 15	dph001	861,0852	1056,72	816
medaka 16	dph001	669,22104	800,68	519,84
medaka 17	dph010	709,2424	1679,4	971,16
medaka 18	dph010	482,869929	1117,28	768,52
medaka 19	dph010	701,1069	1397,6	653,72
medaka 20	dph010	635,3492	1388,88	873,04
medaka 21	dph010	967,202	1589,88	883,72
medaka 22	dph010	472,9577	1435,12	1054,16
medaka 23	dph010	530,706	1036,12	605,04
medaka 24	dph010	674,1712	1358,28	864
medaka 25	dph010	447,27992	1198,04	1092,04
medaka 26	dph010	639,8321	1187,68	972,8
medaka 27	dph010	731,3401	1093,16	908,64
medaka 28	dph010	749,99265	1150,08	1032,52
medaka 29	dph010	774,1093	1477,96	1027,56
medaka 30	dph010	867,53095	985,16	347,32
medaka 31	dph010	237,95409	512,8	837,72
medaka 32	dph010	669,1964	1560,88	861,68
medaka 33	dph030	638,7974	1543,52	1025,72
medaka 34	dph030	320,96726	761	1269,4
medaka 35	dph030	326,62279	579,84	1434,64
medaka 36	dph030	1294,7005	1467,84	1120,16
medaka 37	dph030	732,1774	1578,4	998,84
medaka 38	dph030	1619,4674	1630,28	913,72
medaka 39	dph030	972,2946	1560,28	1111,4
medaka 40	dph030	464,81907	756,8	1495,24
medaka 41	dph030	1100,7267	1498,72	918,04
medaka 42	dph030	1155,0072	1269,72	575,24
medaka 43	dph030	654,0254	1418	917
medaka 44	dph030	1184,8266	1602,64	1056,96
medaka 45	dph030	1348,6093	1487,92	1046,92
medaka 46	dph030	914,5768	1469,84	883,88
medaka 47	dph030	1226,628	1589,16	840,12

medaka 48	dph030	1413,4322	1653,84	896,16
medaka a01	dph120	8853,803	1639,2	444,8
medaka a02	dph120	6095,406	1521,8	138,2
medaka a03	dph120	8983,089	1648	264,4
medaka a04	dph120	5809,6106	1518,8	386,6
medaka a05	dph120	9985,492	1701,8	235,8
medaka a06	dph120	8615,275	1605,2	83,2
medaka a07	dph120	7157,804	1578,8	147,6
medaka a08	dph120	4806,3661	1468,2	302
medaka a09	dph120	10961,951	1719,8	69,4
medaka a10	dph120	8383,358	1599,4	148,8
medaka a11	dph120	6873,607	1591,6	262,4
medaka a12	dph120	6244,906	1658,6	522,2
medaka a13	dph120	11292,124	1729,6	464,2
medaka a14	dph120	7542,592	1618	254,2
medaka a15	dph120	4266,0742	1575,4	463,6
medaka a16	dph120	4043,7819	1530	814,8
